# Effects of Bacillus amyloliquefaciens TL106 Isolated from Tibetan Pigs on Probiotic Potential and Intestinal Microbes in Weaned Piglets

**DOI:** 10.1128/spectrum.01205-21

**Published:** 2022-01-26

**Authors:** Haitao Du, Wangyuan Yao, Muhammad Fakhar-e-Alam Kulyar, Yanmei Ding, Huaisen Zhu, Huachun Pan, Kewei Li, Zeeshan Ahmad Bhutta, Suozhu Liu, Jiakui Li

**Affiliations:** a College of Veterinary Medicine, Huazhong Agricultural University, Wuhan, People’s Republic of China; b Guangxi Yangxiang co., LTD, Guigang City, Guangxi, People’s Republic of China; c The Royal (Dick) School of Veterinary Studies, University of Edinburgh, Midlothian, Scotland, United Kingdom; d College of Animals Husbandry and Veterinary Medicine, Tibet Agricultural and Animal Husbandry University, Linzhi, Tibet, People’s Republic China; Peking University People’s Hospital

**Keywords:** *Bacillus amyloliquefaciens* TL106, weaned piglet, probiotic potential, growth performance, intestinal microbe

## Abstract

Bacillus amyloliquefaciens is a nonpathogenic microorganism whose highly active amylase is widely isolated from soil and plants. TL106 is an isolate of Bacillus amyloliquefaciens isolated from cold- and disease-resistant Tibetan pigs in Linzhi, Tibet. Here, we report that TL106 not only could survive in acidic environments, high bile salt concentrations, and high-temperature conditions but also was resistant to antibiotics. It significantly improved the growth performance of weaned piglets, especially in the prevention of diarrhea. The crude fiber and crude ash digestibility in weaned piglets after TL106 administration was considerably higher than that in other groups. The results of 16S rRNA sequencing conveyed that TL106 stabilized gut microbiota that was disturbed by the weaning process with an increased level of *Lachnospiraceae*, *Peptococcaceae.rc4_4*, *Erysipelotrichaceae.L7A_E11*, and *Mollicutes.RF39*. Hence, this study proved that Bacillus amyloliquefaciens TL106 might be a candidate for antibiotics in Duroc×Landrace×Yorkshire weaned piglets.

**IMPORTANCE** Antibiotics are often used to promote animal growth and prevent diarrhea in weanling piglets. Nevertheless, intestinal pathogenic bacterial resistance and drug residues caused by antibiotic overuse are worthy of concern and demand an urgent solution. Bacillus amyloliquefaciens TL106 has been isolated from cold- and disease-resistant Tibetan pigs in Linzhi, Tibet. It significantly improved the growth performance, decreased diarrhea, increased the absorption of crude substances, and regulated the gut flora homeostasis in Duroc×Landrace×Yorkshire weaned piglets. As an antibiotic candidate, TL106 perfectly displayed its probiotic potential and pollution-free properties.

## INTRODUCTION

The extensive utilization of antibiotics usually helps in promoting growth and preventing diarrhea (1, 2). Nevertheless, intestinal pathogenic bacterial resistance and drug residues caused by the overuse of antibiotics are worthy of attention and need to be resolved (3, 4). Researchers have been concerned about antibiotic substitutes that can improve growth performance and immune function without any biological harm. For this purpose, probiotics have been the greatest advantage in promoting the digestion and absorption of nutrients, improving immunity, maintaining the balance of intestinal flora, and protecting the intestinal mucosal barrier in order to replace antibiotics (5, 6). The Tibetan pigs live at high altitudes, where they face a harsh climate and low temperatures (7, 8), and also have an ability against diseases and crude fiber resistance that allows less exposure to antibiotics than that experienced by other pig breeds (9, 10). Previous studies have shown the excellent characteristics of Tibetan pigs that might be due to the abundant *Bacillus* in their intestines (11, 12).

Bacillus amyloliquefaciens is widely used as a potential probiotic with strong stress resistance characteristics (13–15). It inhibits plant and animal pathogens with excellent inhibitory effect (16–18). Different research reports have found that B. amyloliquefaciens strongly affects plant pathogens, e.g., *Rhizopus*, Trichoderma harzianum CCMI, Botrytis cinerea, *Penicillium* fungi, etc. (16). The *in vitro* study of Larsen et al. has also revealed the antibacterial effects of Bacillus amyloliquefaciens on various animal pathogens, including Escherichia coli, Staphylococcus aureus, and Salmonella (19). Some other studies have also found that B. amyloliquefaciens effectively inhibits the growth of Listeria monocytogenes in meat by secreting bacteriocins (20, 21). European Food Safety Authority (EFSA) reported that Bacillus amyloliquefaciens CECT5940 significantly reduces Clostridium perfringens and E. coli in a chicken’s intestine (18). Moreover, it was also found as a growth promoter in poultry, pig, and aquaculture breeding. According to the research of Zhao et al., the extracellular polysaccharide produced by Bacillus amyloliquefaciens GSBa-1 can be used as a natural antioxidant (22). Cao et al. claimed that adding B. amyloliquefaciens to feed can improve the growth performance of broilers. Some metabolomics analysis also revealed that Bacillus amyloliquefaciens changed the cecal metabolites through the involvement of amino acid and glyceride metabolism (23). Yongtao brings to light that the addition of B. amyloliquefaciens probiotics to the prenatal diet of pregnant sows promoted the production of short-chain fatty acids (SCFAs) in the intestines of both sows and newborn piglets with the improvement of their intestinal flora (24). Moreover, the oral administration of B. amyloliquefaciens SC06 can reduce bacterial translocation and affect the intestinal immune function in weaned mice (25, 26).

In our current study, Bacillus amyloliquefaciens TL106 was isolated from Tibetan pigs (Linzhi, Tibet) and then was used as a feed additive for Duroc×Landrace×Yorkshire weaned piglets to evaluate biological functions. Our findings will be beneficial for animal husbandry with some novel ideas for antibiotic substitutes.

## RESULTS

### Growth of Bacillus amyloliquefaciens TL106 *in vitro*.

**FIG 1:**
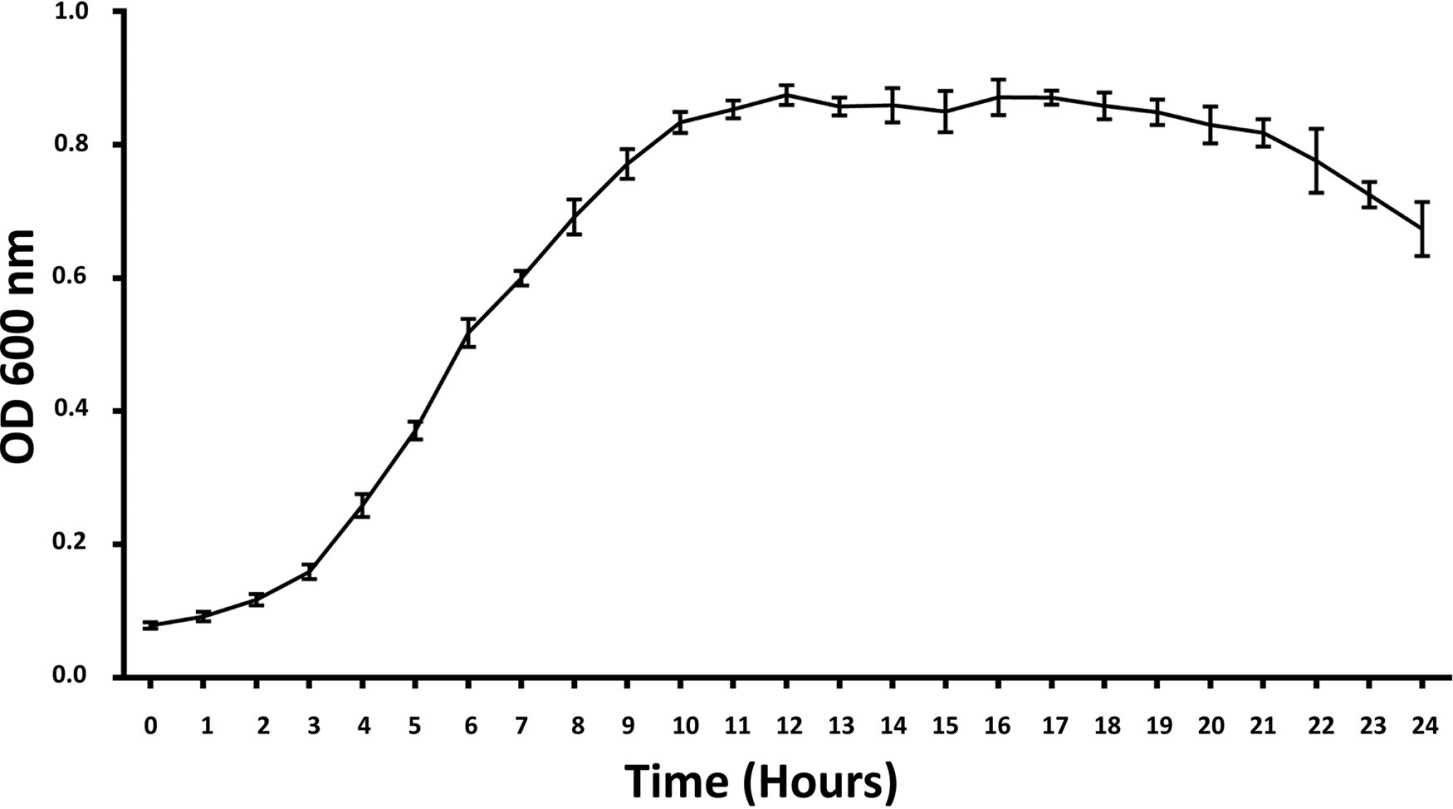
Growth curve of Bacillus amyloliquefaciens TL106 in 24 h. Detection of OD_600_ nm in microplate reader. Repeated five times for each group (*n* = 5).

To examine the growth of Bacillus amyloliquefaciens TL106, we inoculated 1% (vol/vol) of the seeded bacteria liquid into an LB medium. The optical density (OD) value was measured at 600 nm for 24 h on the automatic growth curve analyzer (Bioscreen, Finland). Within 2 h, the OD value changed little, which was the growth lag phase (Fig. 1). Bacillus amyloliquefaciens entered the logarithmic growth phase after 4 h and reached a plateau after 10 h. Although the nutrients and space were limited, the OD value of the bacteria was not significantly decreased after 20 h, indicating that TL106 was stable.

### Stress resistance of Bacillus amyloliquefaciens TL106 *in vitro*.

**FIG 2:**
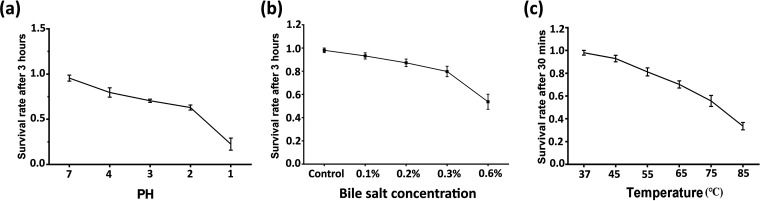
Survival curve of Bacillus amyloliquefaciens under different conditions (pH, bile salt concentration, and temperature). (a) Survival rate of TL106 after 3 h under pH 1 to 4 or 7. (b) Survival rate of TL106 after 3 h under different bile salt concentrations (0.1%, 0.2%, 0.3%, 0.6%) and control. (c) Survival rate of TL106 after 3 h under different temperatures (37°C, 45°C, 55°C, 65°C, 75°C, 85°C). Repeated five times for each group (*n* = 5).

Probiotics must first withstand the internal environment with survival stability to exhibit their functions. Therefore, in the current study, we measured the stress resistance of Bacillus amyloliquefaciens. We simulated the organism’s internal environment with different temperatures, bile salt concentrations, and pH changes. As the pH value decreased (the acidity of the internal environment increased), the survival rate after 3 h of TL106 showed a downward trend. At a pH of 2, the survival rate of probiotics reached 50%. Even under a highly acidic condition (pH = 1), live bacteria were still present in the organism (Fig. 2a).

A high concentration of bile salt posed a significant threat to the survival of probiotics. Bacillus amyloliquefaciens TL106 had a survival rate of 50% at 0.6% bile salt concentration, which showed its strong tolerance ability (Fig. 2b). It is clearly shown in Fig. 2c that the increase in temperature caused a decrease in survival rate after 3 h. When the temperature reached 65°C, the survival rate of TL106 was 60%, decreasing to 30% as temperature reached 85°C. It showed the tenacity of TL106 under such harsh conditions.

### Antibiotic susceptibility of Bacillus amyloliquefaciens and inhibition of pathogenic bacteria.

**FIG 3:**
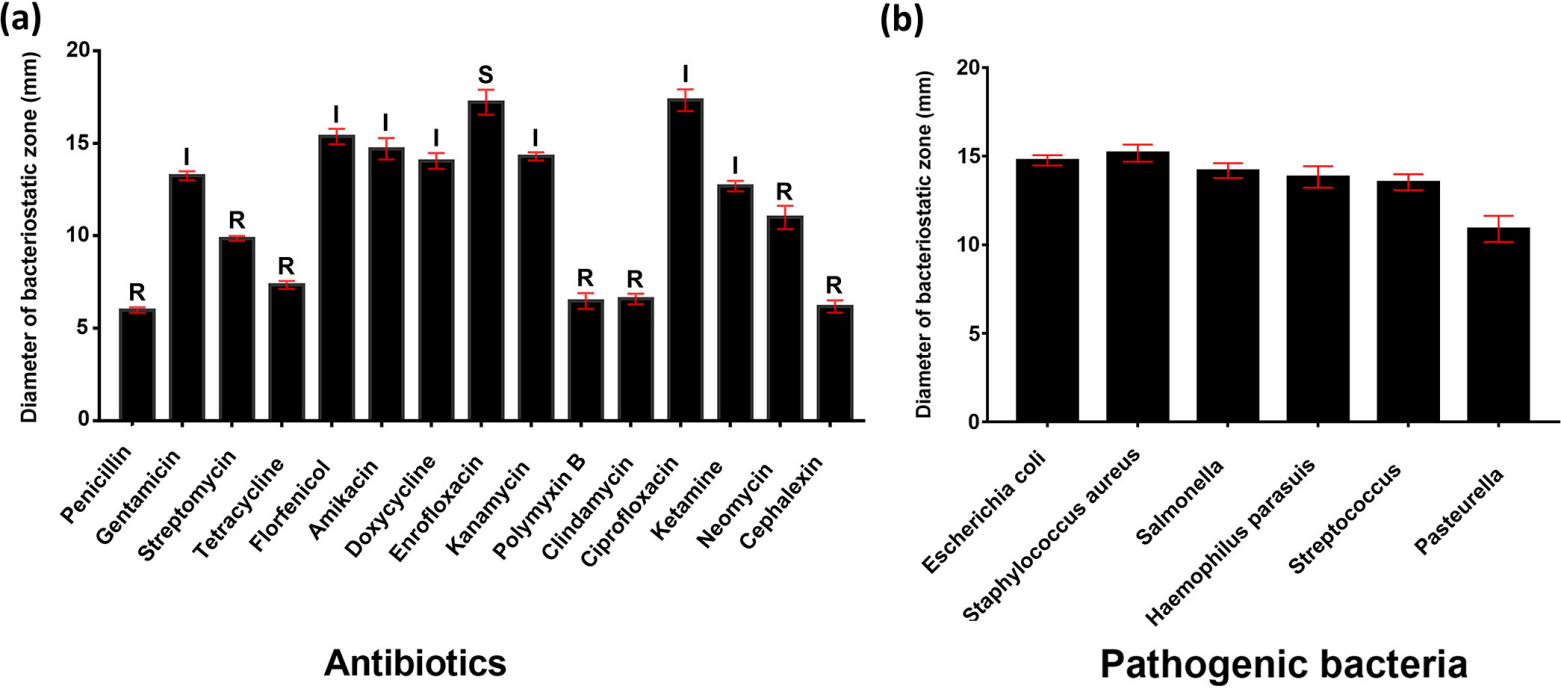
The sensitive survival zone of Bacillus amyloliquefaciens TL106 to antibiotics and pathogens. (a) The diameter of the zone represents the degree of inhibition for antibiotics. (b) The diameter of the zone represents the degree of inhibition for pathogenic bacteria. The diameter of the inhibition zone was measured by vernier caliper and repeated five times for each group (*n* = 5). R, resistance; I, moderately sensitive; S, sensitive.

After tolerating the internal environment, probiotics also need to resist interference from exogenous antibiotics. Under normal circumstances, these antibiotics can eliminate or inhibit pathogenic bacteria in the body and threaten the growth of probiotics to a certain extent. In order to develop the susceptibility of *Bacillus* TL106 to antibiotics, we used 15 antibiotics commonly used in clinical practice. The results directly showed that the diameter of the growth zone inhibited by penicillin, tetracycline, polymyxin B, clindamycin, and cephalexin was relatively small (all within 8 mm). Enrofloxacin and ciprofloxacin had the most potent inhibition on TL106, with the inhibition zone diameter reaching up to 18 mm (Fig. 3a). It is also conveyed that TL106 was very sensitive to quinolone drugs. At the same time, we performed the inhibition of Bacillus amyloliquefaciens TL106 on six pathogenic bacteria. In order to compete with pathogens, growth space and resources were also a crucial part of the probiotic effect of TL106. As shown in Fig. 3b, TL106 had similar inhibition on standard strains of Escherichia coli, Staphylococcus aureus, and Salmonella and clinical strains of Haemophilus parasuis and *Streptococcus*. The diameter of their inhibition zone was between 14 mm and 15 mm. TL106 inhibited clinical strain of *Pasteurella*, which was relatively small, but the size of the inhibition zone was still more than 10 mm in diameter.

### Effect of Bacillus amyloliquefaciens on the growth performance of weaned piglets.

**FIG 4:**
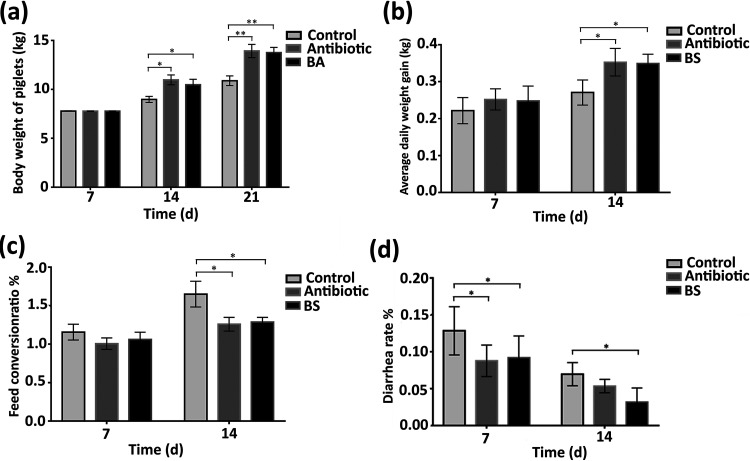
The probiotic effect of Bacillus amyloliquefaciens TL106 on weaned piglets. (a) Carcass weight of three groups of weaned piglets at 7 days and 14 days. (b) The average daily weight gain of the three groups of weaned piglets at 7 days and 14 days. (c) Feed conversion ratio of three groups of weaned piglets at 7 days and 14 days. (d) Diarrhea rate of three groups of weaned piglets at days 7 and 14. Repeated five times for each group (*n* = 5). (*, *P < *0.05; **, *P < *0.01). BA, Bacillus amyloliquefaciens.

The impact on animal growth performance is the primary investigation of probiotics. In this study, we implemented the effect of B. amyloliquefaciens TL106 on weaned piglets. As shown in Fig. 4a, the average piglet weight in the antibiotic and the B. amyloliquefaciens groups increased significantly after weaning on day 7 and day 14 as compared to that in the control group. The weights of the piglets in the probiotic group were not as high as those of piglets in the antibiotic group (*P > *0.05). Also, the average daily weight gain of the piglets during the first 7 days was not significantly different among the three groups. The average daily weight of the antibiotic group was slightly higher than that of the normal and the B. amyloliquefaciens groups. However, between 7 and 14 days, the average daily weight gain of piglets in the antibiotic and B. amyloliquefaciens groups was significantly higher than that of the control group (Fig. 4b). After 7 days, the feed conversion ratio of piglets in the normal and B. amyloliquefaciens groups was slightly higher than that of the antibiotic group, but there was no significant difference (*P > *0.05). After 14 days, compared to that of piglets in the normal group, the feed conversion ratio of the piglets in the antibiotic group and the B. amyloliquefaciens group was significantly lower (Fig. 4c). Diarrhea in weaned pigs is a thorny issue that is a matter of concern for farmers. After 7 days, the administration of antibiotics and Bacillus amyloliquefaciens TL106 significantly reduced piglet diarrhea compared to that in the normal group. The effect of antibiotics was evident at first, but over the course of the 14 days, TL106 was more effective than the antibiotic (Fig. 4d). The above results revealed that TL106 is comparable to antibiotics in probiotic prospects and prevention of diarrhea.

### Effect of Bacillus amyloliquefaciens on the apparent digestion of nutrients in weaned piglets.

**FIG 5:**
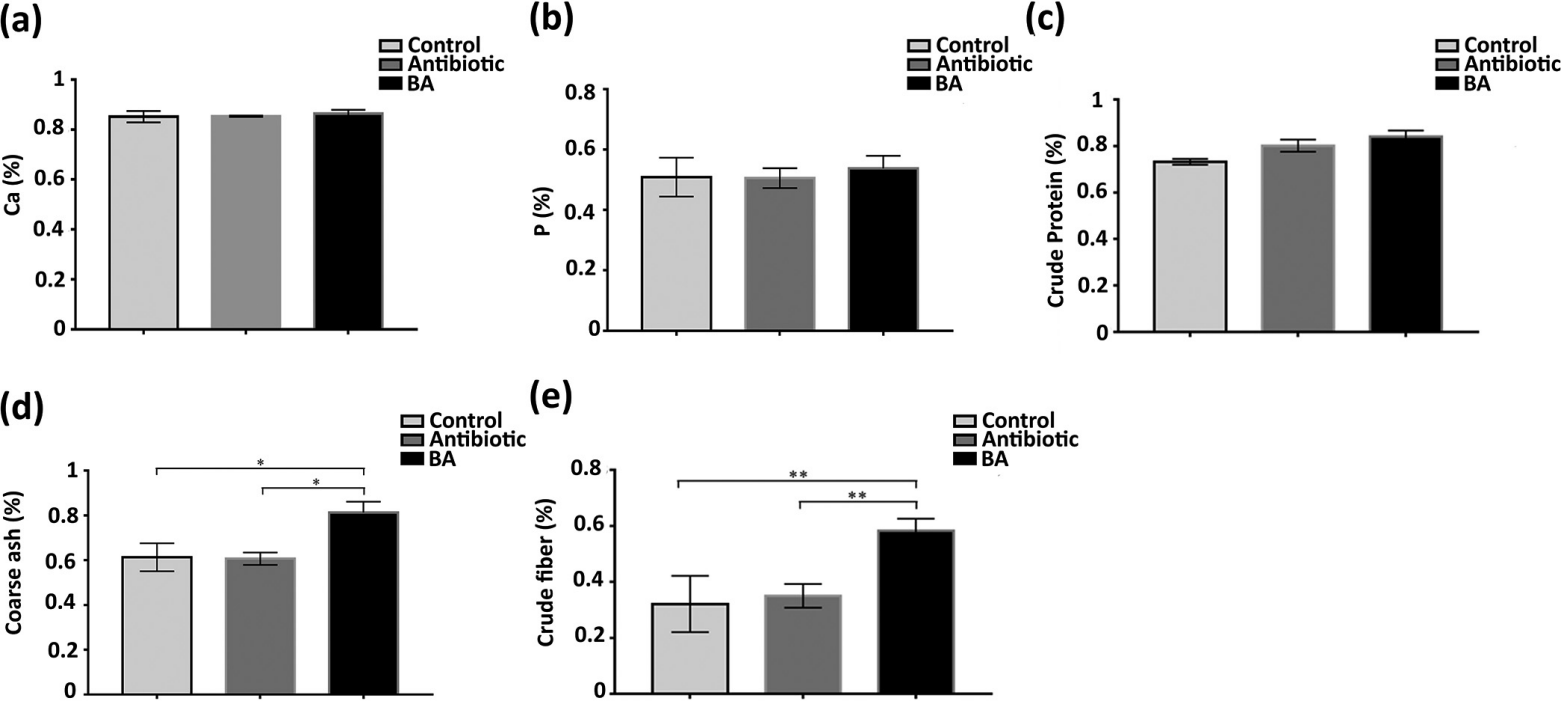
Changes of apparent nutrient digestibility in three groups of weaned piglets. (a) Calcium absorption rate of weaned piglets. (b) Phosphorus absorption rate of weaned piglets. (c) Crude protein absorption rate of weaned piglets. (d) Crude ash absorption rate of weaned piglets. (e) Crude fiber absorption rate of weaned piglets. Repeated five times for each group (*n* = 5). (*, *P < *0.05).

The digestion and absorption of nutrients are essential for weaned piglets. To explore the effect of antibiotic and Bacillus amyloliquefaciens TL106 on the apparent digestibility of nutrients in weaned piglets, we tested several indicators of calcium, phosphorus, crude protein, and crude ash in this study. Although weaned piglets were administered with antibiotics and TL106, there was no difference in the digestibility of calcium, phosphorus, and crude protein compared with that of the control group (Figure 5a to c). In particular, the digestibility of crude ash in the B. amyloliquefaciens group was significantly higher than that in the antibiotic group and the normal group (Fig. 5d). Moreover, the digestibility of crude fiber for weaned piglets in the B. amyloliquefaciens group was significantly higher than that for piglets in the antibiotic group and the normal group. Also, there was no difference between the antibiotic and the normal group (Fig. 5e). These results conveyed that Bacillus amyloliquefaciens TL106 promoted crude ash and crude fiber digestion in weaned piglets.

### Effect of Bacillus amyloliquefaciens on biochemical indexes of weaned piglets.

**FIG 6:**
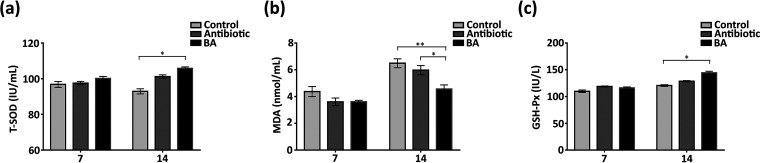
Changes of three biochemical indexes in three different groups of weaned piglets. (a) The level of superoxide dismutase of weaned piglets’ serum in three groups. (b) The level of malondialdehyde in the serum of weaned piglets. (c) The level of glutathione peroxidase in the serum of weaned piglets. Repeated five times for each group (*n* = 5). (*, *P < *0.05; **, *P < *0.01).

Biochemical indexes were detected from weaned piglets to compare the effect of antibiotics and TL106 on the physiological functions of piglets. On the other hand, it was also determined whether or not TL106 has a negative effect on piglets. In the first 7 days, the three groups of weaned piglets were approximately equal, and there was no statistical difference. With increasing age, the total superoxide dismutase (T-SOD) of weaned piglets in the B. amyloliquefaciens group was significantly higher than that of piglets in the normal group. Although the T-SOD and glutathione peroxidase (GSH-Px) in the antibiotic group were higher than those in the normal group, there was statistically no difference on the 14th day (Fig. 6a and c). On the 7th day, the level of malondialdehyde (MDA) of weaned piglets in the normal group was slightly higher than that of weaned piglets in the antibiotic group and the B. amyloliquefaciens group, but the difference was not obvious. However, on the 14th day, the MDA level of weaned piglets in the B. amyloliquefaciens group was significantly lower than that of weaned piglets in the normal and antibiotic groups (Fig. 6b). The above results revealed that Bacillus amyloliquefaciens TL106 could improve weaned piglets’ antioxidant and anti-inflammatory capacity with no side effects on the body.

### Effects of Bacillus amyloliquefaciens on the intestinal digestive enzyme activity.

**FIG 7:**
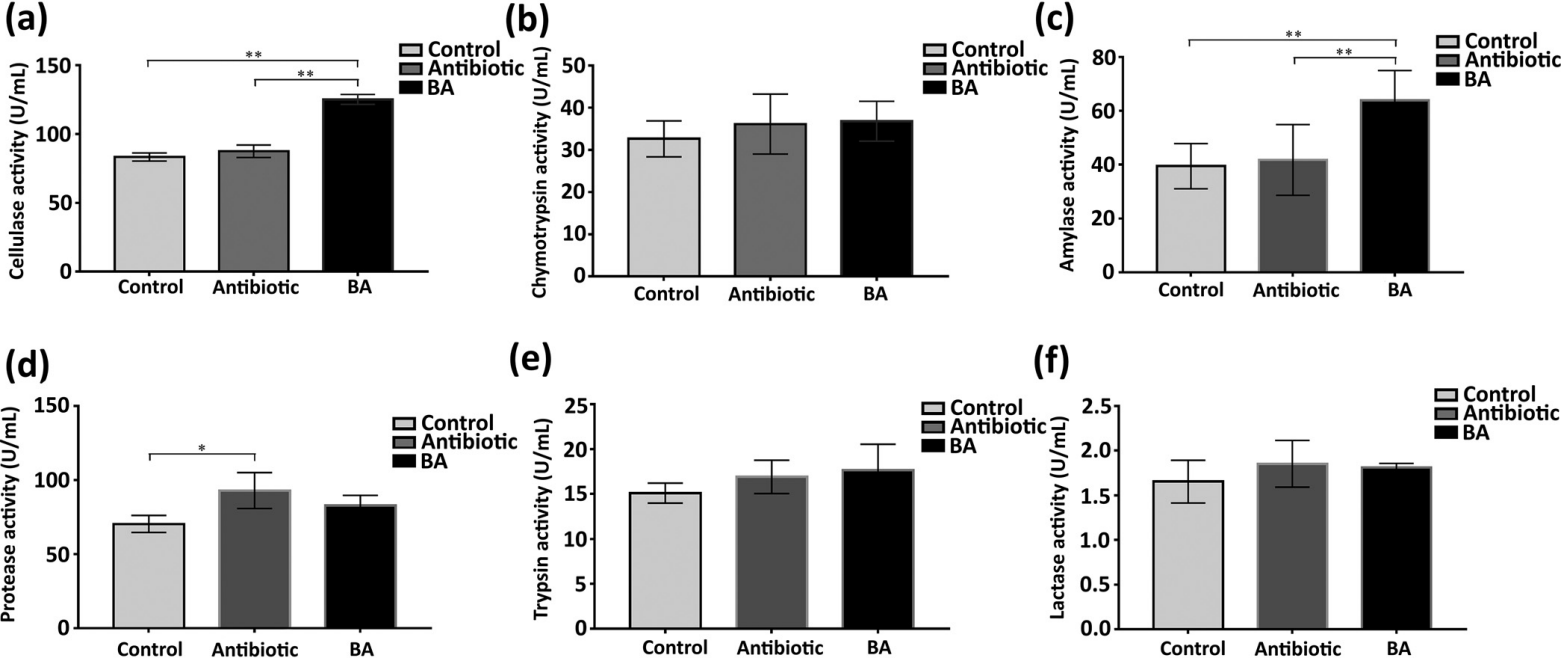
Changes of digestive enzymes in three groups of weaned piglets. (a) The levels of cellulase activity in three groups. (b) The level of chymotrypsin activity in the three groups. (c) The level of amylase activity in the three groups. (d) The level of protease activity in the three groups. (e) The level of trypsin activity in the three groups. (f) The level of lactase activity in the three groups. Repeated five times for each group (*n* = 5). (*, *P < *0.05; **, *P < *0.01).

Most of the substances are digested and absorbed in the small intestine of piglets, and various digestive enzymes are also present here. Therefore, we evaluated the effect of TL106 on the intestinal digestive enzyme (cellulase, chymotrypsin, amylase, protease, trypsin, lactase) activity of ileum content. As shown in Fig. 7a, c, and d, there were significant differences in cellulase activity, amylase activity, and protease activity among the three groups. Compared with those in the normal and antibiotic groups, cellulase activity and amylase activity were significantly enhanced in the TL106 groups. However, compared with that in the normal group, the protease activity was significantly enhanced in the antibiotic group. Although the probiotic group had lower protease activity than the antibiotic group, there was no statistical difference. Chymotrypsin activity and trypsin activity in the probiotic group were slightly higher than those in the normal and antibiotic groups, but they were not as significant. The above results indicated that Bacillus amyloliquefaciens TL106 significantly improved the activity of digestive enzymes, especially cellulase and amylase. There was a possibility that TL106 could promote the growth of weaned piglets and promote the digestion and absorption of intestinal nutrients.

### Analysis of the colonization of Bacillus amyloliquefaciens TL106 in the intestine.

**FIG 8:**
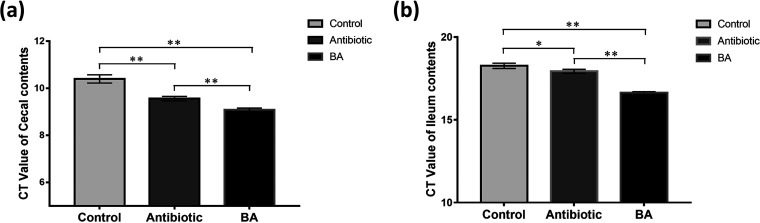
The colonization of Bacillus amyloliquefaciens TL106 in the intestinal contents of weaned piglets in three groups by RT-qPCR. (a) RT-qPCR results (*C_T_* value) of the cecal contents in three groups. (b) RT-qPCR results (*C_T_* value) of the ileum contents in three groups. Repeated five times for each group (*n* = 5). (*, *P < *0.05; **, *P < *0.01).

The clinical manifestations of weaned piglets showed that Bacillus amyloliquefaciens had been active in the piglets’ intestines. In order to further determine the colonization of TL106 in the intestine, we performed reverse transcriptase quantitative PCR (RT-qPCR) for verification. We extracted total DNA from the contents of the cecum and ileum of weaned piglets in the three groups and adjusted the DNA concentration to the same level. According to the principle of RT-qPCR, in the same system, the larger the cycle threshold (*C_T_* value), the lower the content of TL106. On the contrary, the smaller the cycle threshold (*C_T_* value), the greater the content of TL106. As shown in Fig. 8a, compared to that in the control and antibiotic groups, the *C_T_* value of the cecal contents in the B. amyloliquefaciens group was significantly lower. It also means that the content of Bacillus amyloliquefaciens TL106 in the cecum of weaned piglets in the B. amyloliquefaciens group was significantly higher than that in the control group and the antibiotic group. The *C_T_* value of ileum content in the control and antibiotic groups was significantly greater than that in the B. amyloliquefaciens groups. Therefore, the content of Bacillus amyloliquefaciens TL106 in the ileum of weaned piglets in the B. amyloliquefaciens group was substantially higher than that in the control group and the antibiotic group (Fig. 8b). Based on the above results, it was determined that Bacillus amyloliquefaciens TL106 was colonized in the intestines of piglets in the B. amyloliquefaciens group, but the content of TL106 in the B. amyloliquefaciens group was significantly higher than that in other groups. In addition, the content of TL106 in the cecum was more than that in the ileum.

### Effects of Bacillus amyloliquefaciens on inflammatory factors and immunoglobulins levels in serum of weaned piglets.

**FIG 9:**
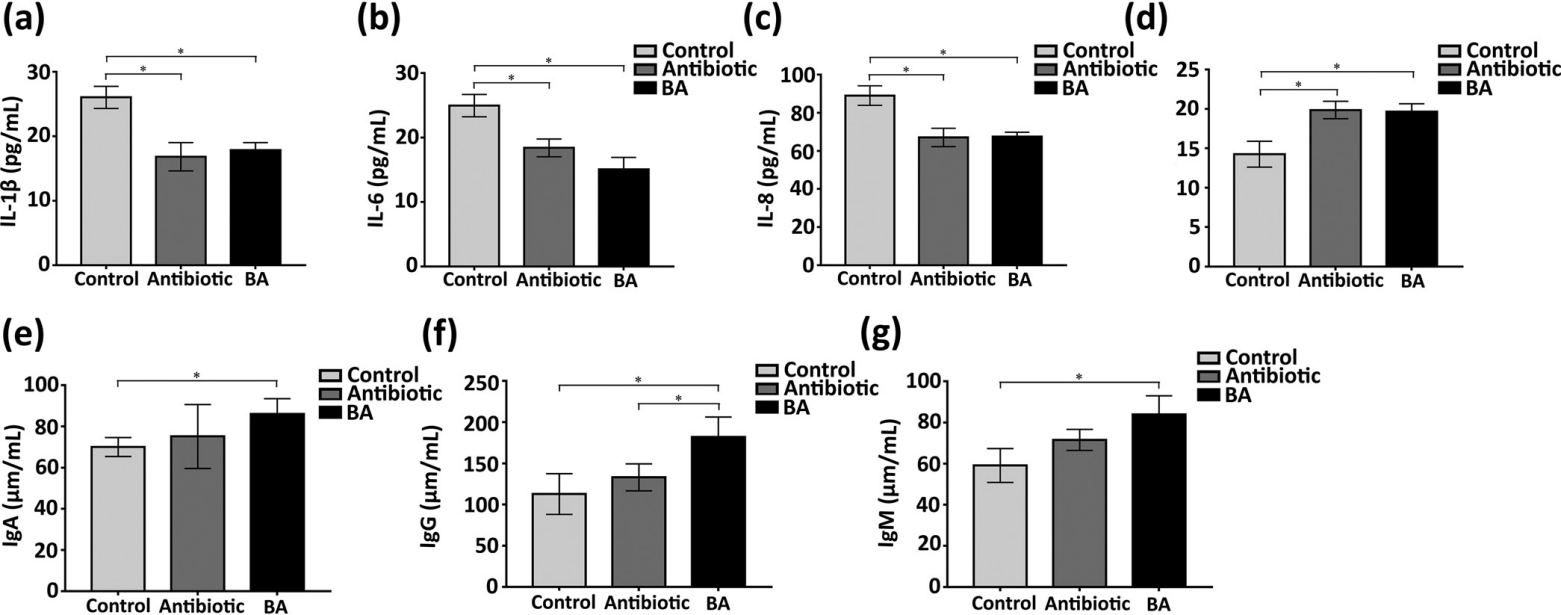
Changes of serum inflammatory factors and immunoglobulins in three groups of weaned piglets. (a to d) The levels of IL-1β, IL-6, IL-8, and IL-10 of serum in three groups. (e to g) The levels of IgA, IgG, and IgM of serum in three groups. Repeated five times for each group (*n* = 5). (*, *P < *0.05).

To evaluate whether TL106 could relieve inflammatory response and weaken immunity caused by weaned stress, we examined the inflammatory cytokines interleukin 1β (IL-1β), IL-6, IL-8, and IL-10 and immunoglobulins IgA, IgM, and IgG in the serum of weaned piglets. As shown in Fig. 9a to d, levels of proinflammatory cytokines IL-1β, IL-6, and IL-8 were higher in the serum of the control group than those in the B. amyloliquefaciens group (*P < *0.05) and levels of anti-inflammatory cytokine IL-10 were lower (*P < *0.05). Although these indicators fluctuated up and down in the antibiotic and B. amyloliquefaciens groups, there was no statistical difference. Next, we checked the level of immunoglobulins in the serum of weaned piglets. The results intuitively conveyed that the levels of IgA, IgG, and IgM in the serum of the B. amyloliquefaciens group were significantly higher than those in the normal group; in particular, the IgG in the serum of the B. amyloliquefaciens group was also significantly higher than that of the antibiotic group. The level of immunoglobulins in the antibiotic group was slightly higher than that in the normal group, but there was no statistical difference (*P > *0.05) (Fig. 9e to g). These results preliminarily proved that the weaned stress could cause the increase of serum inflammatory factors and decrease immunoglobulin, which can be eliminated through Bacillus amyloliquefaciens TL106.

### Effect of Bacillus amyloliquefaciens on the immune organs (spleen, mesenteric lymph nodes) of weaned piglets.

**FIG 10:**
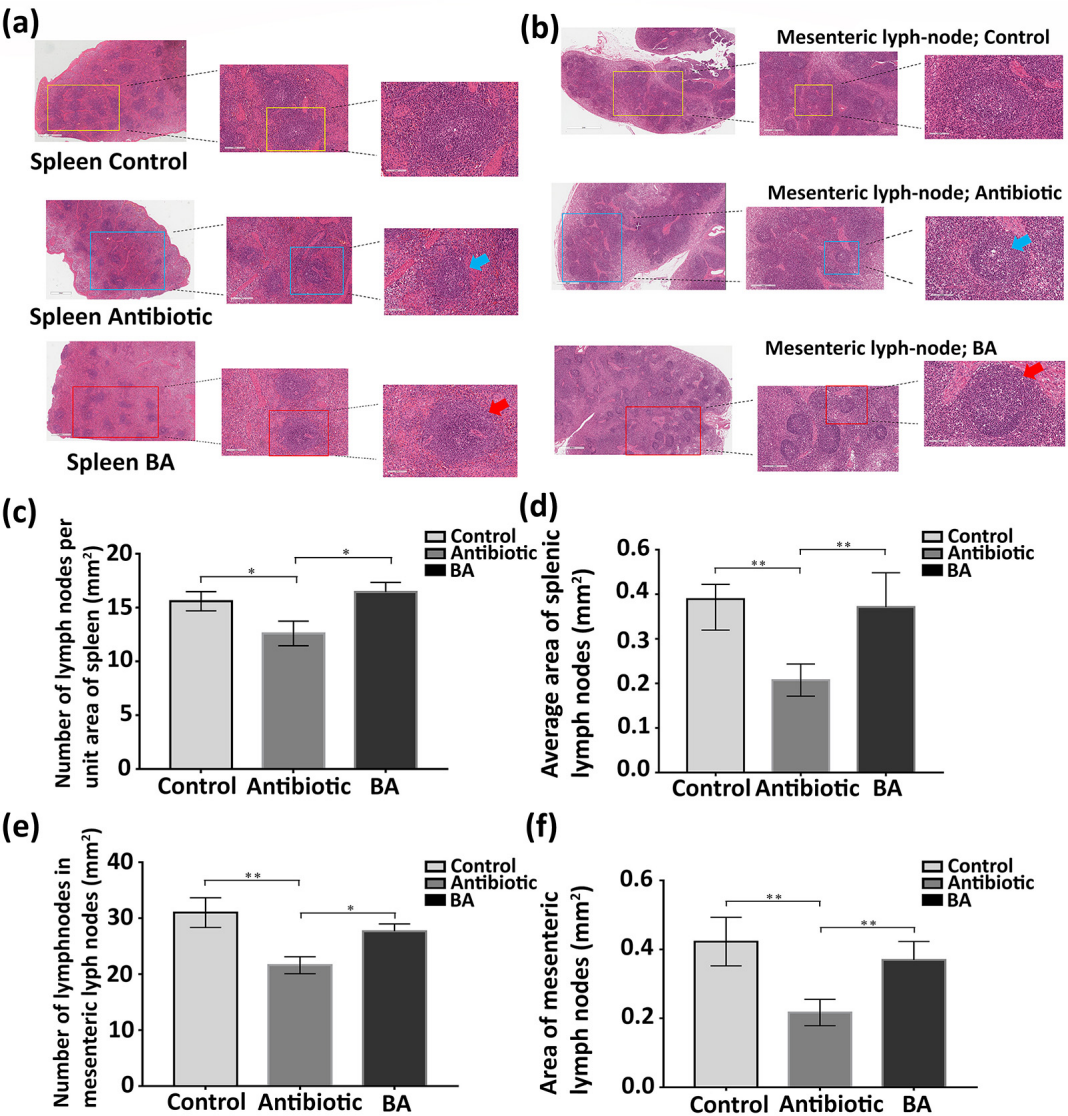
H&E staining of spleen and mesenteric lymph nodes of weaned piglets. (a) Pathological changes of spleen in three groups of weaned piglets. (b) Pathological changes of mesenteric lymph nodes in three groups of weaned piglets. (The scale of the figures from left to right is 1 mm, 200 μm, 100 μm.) (c) Number of lymph nodes per unit area of spleen in three groups. (d) Average area of splenic lymph nodes in three groups. (e) Number of lymph nodes per unit area of mesenteric lymph node in three groups. (f) Average area of the lymph nodes of the mesenteric lymph nodes in three groups. Repeated five times for each group (*n* = 5). (*, *P < *0.05; **, *P < *0.01).

To examine the effect of antibiotics and B. amyloliquefaciens on the tissue structure of the immune organs of weaned piglets, we randomly selected five pigs from each group and performed hematoxylin and eosin (H&E) staining of pathological sections of spleen and mesenteric lymph nodes. As shown in Fig. 10a, the structure of the splenic lymph nodes in the same area in the antibiotic group was blurred, and the area of the lymph nodes became smaller compared with that of the lymph nodes in the control group. However, there was no significant difference between the splenic lymph nodes in the normal group and those in the B. amyloliquefaciens group. Like the observation results of the spleen, the number and surface area of the lymph nodes of mesenteric lymph nodes were reduced in the antibiotic group (Fig. 10b). Next, we digitized the content of the picture with each group of five different piglets of the same tissue sections. We used the same unit area to count the number of lymph nodes and then calculated the lymph nodes’ surface area with software (Aperio ImageScope). Figure 10c and d revealed that not only was the number of lymph nodes in the antibiotic group significantly smaller than that in the other two groups (*P < *0.05) but the surface area of lymph nodes had also decreased (*P < *0.01). We also found that, compared to that in the B. amyloliquefaciens group, the difference was more significant than that in the normal group (*P < *0.01).

### The effect of Bacillus amyloliquefaciens on the digestive organs (duodenum, jejunum, ileum) of weaned piglets.

**FIG 11:**
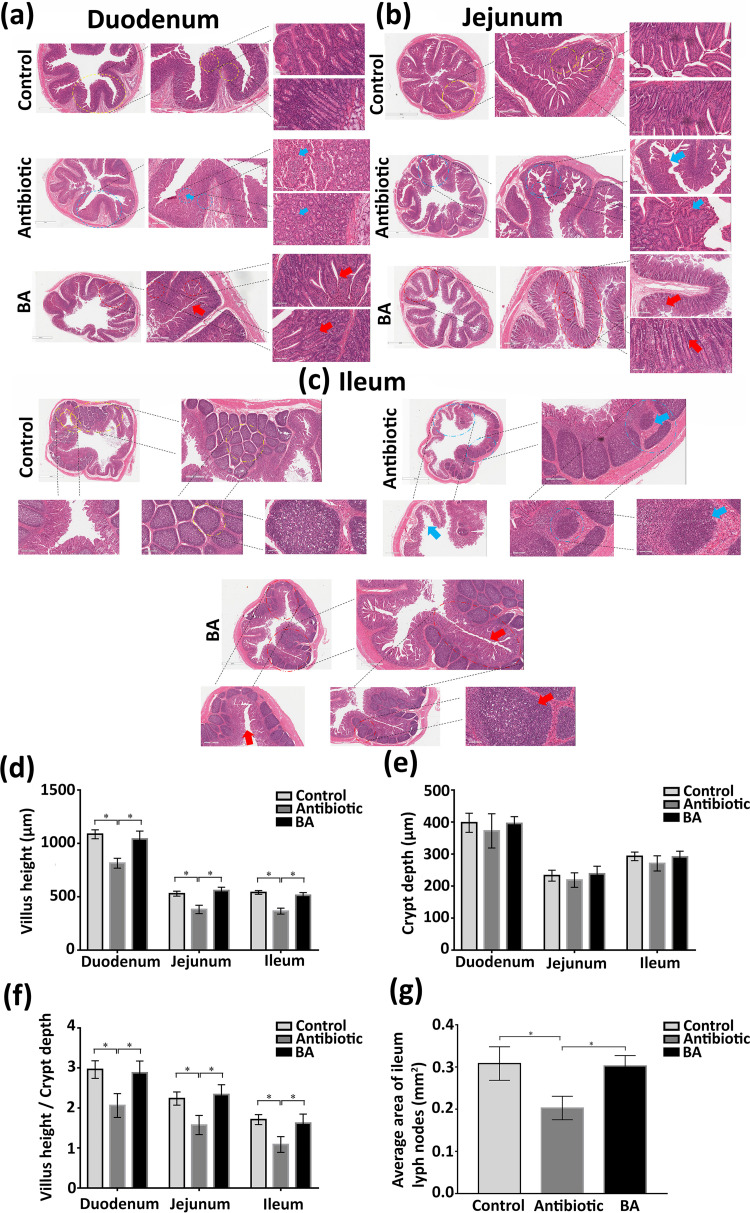
H&E staining of duodenum, jejunum, and ileum of weaned piglets. (a) Pathological changes of duodenum in three groups of weaned piglets. (b) Pathological changes of jejunum in three groups of weaned piglets. (c) Pathological changes of ileum in three groups of weaned piglets. (d) Villi height of duodenum, jejunum, and ileum in three groups. (e) Crypt depth of duodenum, jejunum, and ileum in three groups. (f) The ratio of the villi height of the duodenum, jejunum, and ileum to the crypt depth in three groups. (g) The average area of ileum lymph nodes in three groups. (The scale of the figures from left to right is 1 mm, 200 μm, 100 μm). Repeated five times for each group (*n* = 5). (*, *P < *0.05).

To demonstrate the effect of TL106 on the structure and function of the digestive system, we used the normal group and the antibiotic group as references. As shown in Fig. 11a to c, the intestinal villi of the duodenum, jejunum, and ileum in the antibiotic group piglets were arranged irregularly. The villi structure was imperfect and even fragmented. In addition, in the antibiotic group, the distribution of intestinal epithelial goblet cells was disordered, and the number of goblet cells was also decreased. However, the B. amyloliquefaciens group’s intestinal wall and villi were intact and arranged evenly and orderly, the same as those of the control group (Figure 11a to c).

Next, we digitized the content of the pictures of the same tissue sections of five different piglets from each group. As shown in Fig. 11d, compared with those in the control and the B. amyloliquefaciens groups, the villus heights of the duodenum, jejunum, and ileum in the antibiotic group decreased significantly (*P < *0.05). Although the crypt depth of the duodenum, jejunum, and ileum in the antibiotic group was lower than that in the control group and the B. amyloliquefaciens group, there was no statistical difference between the three groups (Fig. 11e). The average area of lymph nodes in each group was also analyzed. The average area of ileum lymph nodes in the antibiotic group was significantly smaller than that in the normal and B. amyloliquefaciens groups. There was no significant difference between the normal group and the B. amyloliquefaciens group (Fig. 11g).

### Effect of Bacillus amyloliquefaciens on the intestinal microbes of weaned piglets.

**FIG 12:**
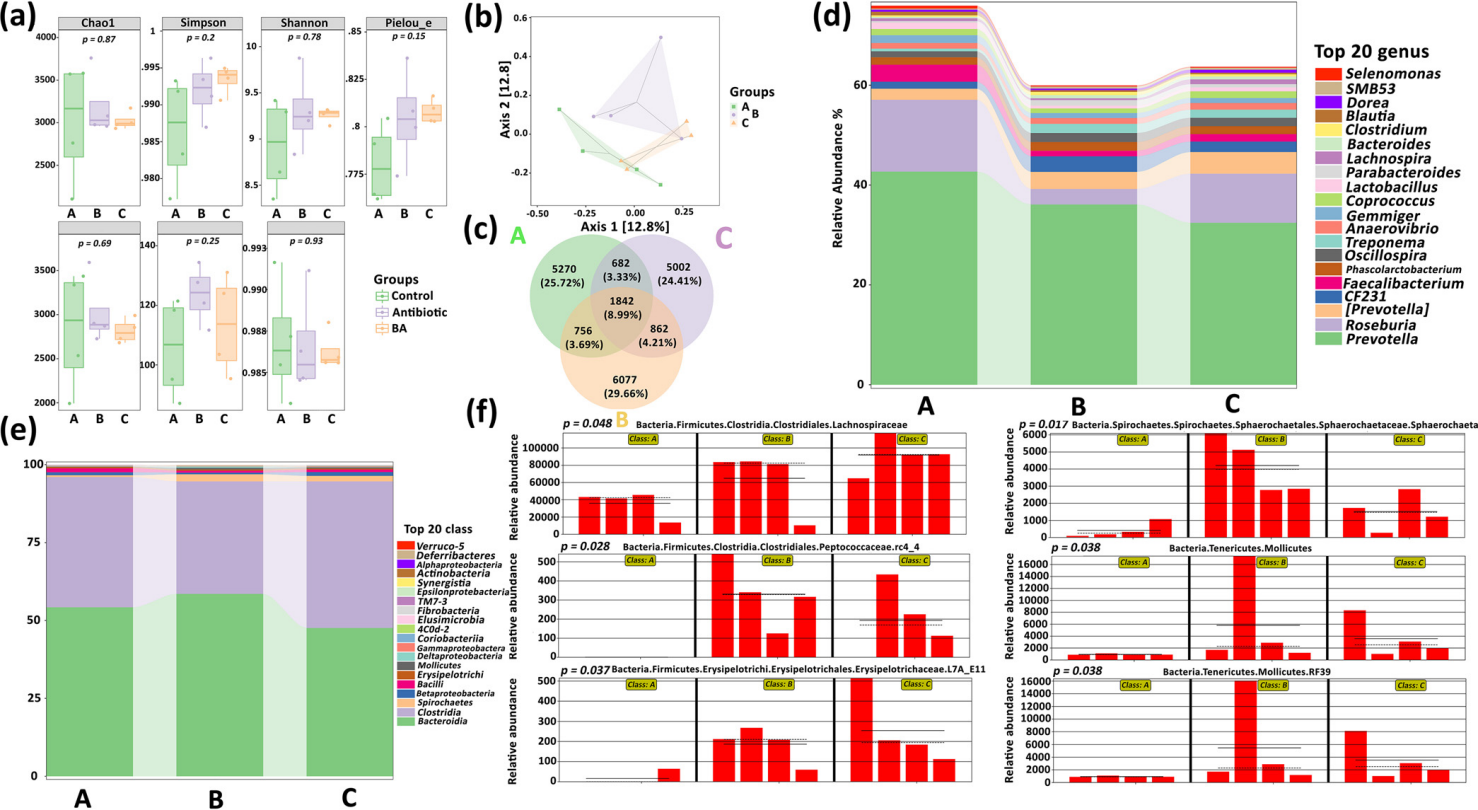
Analysis of intestinal microbial diversity composition of weaned piglets based on 16S rRNA sequencing. (a) Intestinal microbial alpha diversity index of weaned piglets in three groups. (b) PCoA of beta diversity of intestinal microbes of weaned piglets in three groups. (c) Analysis of ASV/OTU Venn diagram of intestinal microbial community of weaned piglets in three groups. (d) Analysis of the abundance of intestinal microbes of weaned piglets at the phylum level classification in three groups. (e) Analysis of the abundance of intestinal microbes of weaned piglets at the genus level classification in three groups. (f) Analysis of intestinal microbes of weaned piglets in three groups for species differences and markers through Linear discriminant analysis (LDA) effect size.

Finally, to evaluate the effect of TL106 on the intestinal microbial diversity of weaned piglets, the 16S rRNA sequencing was performed to analyze the cecal contents in the three groups. We have processed the results of sequencing with different analysis methods and different ways, e.g., alpha diversity. To more comprehensively evaluate the alpha diversity of the microbial community, Chao1 was used. The species indexes were observed to characterize the richness, Shannon index, and Simpson index; Faith’s phylogenetic diversity (PD) index represents evolution-based diversity, Pielou’s evenness index represents uniformity, and Good’s coverage index represents coverage. From the box plot, we observed that the microbial richness of the control group was higher than that of the other groups; also, the microbial diversity of the B. amyloliquefaciens group was, overall, higher. The evolutionary diversity of the antibiotic group was slightly higher than that of the control group and the B. amyloliquefaciens group. Unfortunately, there was no statistical difference between the groups. However, it was easy to see that the B. amyloliquefaciens group’s microbiological data distribution was highly consistent, while those of the control and antibiotic groups were low. We simultaneously obtained the same results in the distance matrix of the beta diversity analysis and the principle-coordinate analysis (PCoA). Compared with those of the control and the antibiotic group, the four data in the B. amyloliquefaciens group were relatively concentrated and closed (Fig. 12a and b). Next, a Venn diagram was used for community analysis to explore which species were shared among different samples (groups) and which were unique. The amplicon sequence variant/operational taxonomic unit (ASV/OTU) value in the antibiotic group was 6,077 and accounted for 29.66%, higher than that in the control and B. amyloliquefaciens groups (Fig. 12c). There was no significant difference between the three groups.

The specific composition of the microbial community at each level of classification was essential for evaluating the function of antibiotics and probiotics. Here, we displayed and analyzed the abundance of the components of only at the phylum and the genus levels the top 20 microbial communities at the phylum and the genus levels. The abundance of species composition at the genus level in Fig. 12d shows that the top five abundances in the three groups were *Selenomonas*, *SMB53*, *Dorea*, *Blautia*, and *Clostridium*. The abundance was the highest in the control group and the lowest in the antibiotic group. *Phascolarctobacterium*, *Faecalibacterium*, *CF231*, *Roseburia*, and *Prevotella* have the lowest abundance in the three groups. Unlike the above, *Prevotella* had the lowest abundance in the B. amyloliquefaciens group.

Similarly, we also analyzed the species abundance of each group at the phylum level in Fig. 12e. The top five abundances of the three groups were *Verruco-5*, *Deferribacteres*, *Alphaproteobacteria*, *Actinobacteria*, and *Synergistia*, but their abundances were similar among the three groups. Intragroup analysis showed that the abundance of *Bacteroidia* was the lowest among the three groups, while intergroup analysis showed that the abundance of *Bacteroidia* was the lowest in the B. amyloliquefaciens group and the highest in the control group. Importantly, we directly analyzed the differences of all classification levels by linear discriminant analysis effect size (LEfSe). At the same time, LEfSe focused on finding biomarkers among the groups (Fig. 12f). The B. amyloliquefaciens group’s abundance of *Bacteria*, *Firmicutes*, *Clostridia*, and *Lachnospiraceae* was significantly higher than that of the control and antibiotic groups (*P < *0.01). *Bacteria*, *Firmicutes*, *Clostridia*, and *Peptococcaceae.rc4_4* were not found in the normal group, but they existed in the antibiotic and B. amyloliquefaciens groups; also, the abundance of antibiotic groups was higher than that of the B. amyloliquefaciens group (*P < *0.01). Compared to that of the antibiotic and B. amyloliquefaciens groups, the abundance of *Bacteria*, *Firmicutes*, and *Erysipelotrichaceae.L7A_ E11* in the control group was notably lower (*P < *0.01), while the abundance of *Bacteria*, *Spironchaetes*, *Sphaerochaetales*, *Sphaerochaeta*, *Tenericutes*, and *Mollicutes.RF39* in the antibiotic group was significantly higher than that in the control and B. amyloliquefaciens groups, and the abundance of the control group was the lowest (*P < *0.01).

## DISCUSSION

The weaning of piglets causes a decrease in growth performance, immune system, and intestinal flora with a high level of weaning stress (27, 28). Piglet diarrhea is an intractable disease easily caused by changes in the feeding environment and intestinal environment after weaning. It is one of the major causes of economic losses in the pig industry (29). Although antibiotics played a key role in this period, the emergence and development of probiotics gradually eliminated our excessive dependence on antibiotics (30). The purpose of this study was to evaluate the probiotic potential of TL106 for Duroc×Landrace×Yorkshire weaned piglets to find a safe and effective candidate to replace antibiotics. We demonstrated that TL106 qualified the probiotic potential of strong stress resistance, growth promotion, and roughage absorption without significant tissue damage. More meaningfully, the results conveyed that TL106 can significantly reduce the diarrhea of weaned piglets, effectively enhances immunity, and improves the stability of the intestinal flora.

The vital role of probiotics is to promote the physiological health of the host by surviving in the intestines (31). Therefore, the isolated strain must survive under low pH and high bile salt concentrations (32). Also, these should be resistant to external antibiotic interference and have competency for survival resources with endogenous bacteria in the piglets (33). According to the current study, the number of viable Bacillus subtilis cells reduced to 10% of the original number, after 18 min at 98°C. At 105°C, this process shortened to 1.4 min. In contrast, the number of viable bacteria in B. amyloliquefaciens reduced to one-tenth of the original number for 2 min at 105°C (34). Larsen et al. also found that the survival rate of B. amyloliquefaciens under high-temperature conditions was significantly higher than that of Bacillus subtilis, Bacillus licheniformis, Bacillus pumilus, and Bacillus mohaiwei (19). Bacillus amyloliquefaciens can also tolerate antibiotics such as penicillin and ampicillin. It has a strong tolerance to pepsin and bovine bile. Our research also proved that B. amyloliquefaciens TL106 had a survival rate under low acid, high bile salt, and high temperature conditions. It was resistant to antibiotics such as penicillin, streptomycin, tetracycline, polymyxin B, and clindamycin. EFSA reported that Bacillus amyloliquefaciens CECT5940 can significantly reduce the number of Clostridium perfringens and E. coli cells in the chicken intestine (18). Larsen et al. found through *in vitro* tests that Bacillus amyloliquefaciens had antibacterial effects on a variety of animal pathogens, including Escherichia coli, Staphylococcus aureus, and *Salmonella* (19). Compaoré et al. demonstrated that Bacillus amyloliquefaciens subsp. can effectively inhibit most *Salmonella* species (35). Studies have also shown that Bacillus amyloliquefaciens has broad-spectrum antibacterial effects on Gram-positive and Gram-negative bacteria (36). Here, we observed that the TL106 inhibited the growth of standard strains of Escherichia coli, Staphylococcus aureus, and *Salmonella in vitro* (inhibition zone diameter 15 mm). In particular, we discovered for the first time that TL106 can also inhibit the clinical strains of Haemophilus parasuis and *Streptococcus*, but the inhibitory effect on *Pasteurella* was mediocre.

Bacillus amyloliquefaciens has been confirmed as a direct edible strain by the U.S. Food and Drug Administration (37) because it does not produce toxic metabolites. It was recognized as a safe bacterium by the European Food Safety Agency (15). *Bacillus* species can survive in intestinal tracts under harsh conditions with the characteristics of forming bacterial membranes, secreting growth-promoting and antibacterial metabolites, etc. (38, 39). The results of Ahmed et al. showed that the addition of 20 g/kg Bacillus amyloliquefaciens in the diet significantly increased the average daily feed intake and daily weight gain of broilers (40). Lei et al. confirmed that the addition of different doses of Bacillus amyloliquefaciens in the diet significantly improved the overall growth performance and morphology of small intestine and increased the number of lactobacilli in the cecum (41). Ji, Hu, and Li reported that Bacillus amyloliquefaciens SC06 significantly increased the daily weight gain and reduced the diarrhea rate of weaned piglets *in vivo* (26). In our study, TL106 and antibiotics significantly increased the bodyweight of piglets at 7 and 14 days after weaning. After 14 days, the daily gain increased, and the feed conversion ratio decreased significantly. We were surprised that antibiotics and TL106 significantly inhibited the diarrhea rate of piglets after 7 days of weaning, but the effect of TL106 was better than that of antibiotics after 14 days. Ridha and Azad reported that feeding Bacillus amyloliquefaciens isolated from fish significantly increased the content of lysozyme, immunoglobulin, and superoxide dismutase in tilapia serum (42). In the process of metabolism, *Bacillus* secreted cellulase, α-amylase, p-glucanase, protease, lipase, xylanase, phosphatase, and other enzymes to supplement the deficiency of endogenous digestive enzymes in animals and improved the digestion and absorption capacity of intestinal tract for nutrients (43). We established that the digestion and absorption of crude ash and crude fiber were significantly increased in the TL106 group. Moreover, with the administration of TL106, the cellulase activity and amylase activity of weaned piglets improved significantly. TL106 not only improved the antioxidant capacity of weaned piglets but also significantly improved its own immunity in our study. This evidence may indicate that antibiotics focus on the surface only through the drug itself, but TL106 works by improving the intrinsic function of weaned piglets. At the same time, we also confirmed that antibiotics had a negative effect on the structure and function of intestinal digestive, absorptive, and immune organs of weaned piglets. However, TL106 played an optimistic role without causing any harm to piglets.

Significant changes occurred in the intestinal microbes of piglets after weaning, manifested mainly as the decrease in the diversity of bacterial communities and the changes in the composition of the bacterial colonies (44, 45). Some studies suggested that the weaning process reduced the diversity of intestinal microbes in healthy piglets by reducing the number of lactobacilli and increasing the proportions of Escherichia coli, *Shigella*, and *Acetobacter* in intestines that further resulted in an imbalance of intestinal flora, leading to diarrhea of piglets (46, 47). It was found that Bacillus amyloliquefaciens *fsznc-06* and Bacillus pumilus *fsznc-09* regulated microbial community by increasing the abundance of beneficial bacteria but decreasing the abundance of pathogenic bacteria in rumen and cecum in weanling black goats (48). Bao et al. reported that TL106 stabilized gut microbiota, disturbed by increased and decreased level of different bacterial colonies, e.g., O157:H7, *Lachnospiraceae*, *Prevotellaceae*, *Muribaculaceae*, *Akkermansiaceae*, and *Lactobacillaceae* in mice (49). In our study, antibiotics and TL106 had no significant effect on the intestinal flora diversity of weaned piglets, although other studies reported that TL106 significantly increased the diversity of intestinal flora. Here, we confirmed that the intestinal flora of weaned piglets in the TL106 group was more stable compared to that of the weaned piglets in the normal group and the antibiotic group. It also reflected the beneficial effect of TL106 in developing and regulating intestinal flora in weaned piglets. Also, we noticed a higher abundance of *Bacteroides*, *Blautia*, *Dorea*, *Escherichia*, and *Fusobacterium* before weaning, while *Prevotella* and *Clostridium* were noticed to be more abundant after weaning (50). Our current study demonstrated the abundance of intestinal *Lachnospiraceae*, *Peptococcaceae.rc4_4*, and *Erysipelotrichaceae.L7A_E11* in weaned piglets that increased significantly with administration of TL106. Many other studies have also found that *Lachnospiraceae* and *Peptococcaceae* are important for the butyrate producers in gut microbiota. Butyrate and other short-chain fatty acids inhibit intestinal inflammation through different mechanisms, maintain the intestinal barrier, and regulate intestinal motility (51–54). *Erysipelotrichaceae* belongs to *Firmicutes* and plays a vital role in host physiology and pathology, including inflammation and lipid metabolism. Duan et al. found that the level of *Erysipelotrichaceae* in the intestines of patients with relapsed Crohn’s disease was significantly lower, and Papadopoulos et al. observed a decrease in the abundance of *Erysipelotrichales* in patients with new-onset Crohn’s disease (53, 54). Hence, TL106 positively might be a competitive approach for intestinal inflammation, intestinal immune barrier, and nutrient metabolism by regulating the intestinal flora in weaned piglets.

### Conclusion.

This study showed that Bacillus amyloliquefaciens TL106 from Tibetan pigs improved growth performance, reduced the rate of diarrhea, and enhanced immunity in Duroc×Landrace×Yorkshire weaned piglets. It also regulated microbial communities by increasing the richness of beneficial bacteria and decreasing pathogenic bacteria for intestinal homeostasis, immune regulation, and nutrient absorption.

## MATERIALS AND METHODS

### Experimental materials.

TL106 samples isolated from Tibetan pigs’ feces were stored in China General Microbiological Culture Collection Center (no. CGMCC 20391) (55). Duroc×Landrace×Yorkshire weaned piglets were raised and tested in the northeast area of Yangxiang Co., Ltd. Bacterial culture medium was purchased from Qingdao Hope Bio-Technology Co., Ltd. Antibiotic susceptibility tablets were purchased from Hangzhou Microbial Reagents Co., Ltd. The serum biochemical kits were purchased from Nanjing Jiancheng Bio Engineering Institute. Oxytetracycline calcium was produced in Jiangxi Xingding Technology Co., Ltd. Porcine bile salt was purchased from Sigma-Aldrich. The 4% formaldehyde fixing solution was purchased from Biosharp Life Sciences (product no. BL539A).

### Experimental design.

All procedures were approved and performed by the Laboratory Animals Research Centre of Hubei, Liaoning, and Tibet, People’s Republic of China under the consent of the ethics committee of Huazhong Agricultural University, People’s Republic of China (permit no. 4200695757). Moreover, all animal experiments and procedures were conducted under the relevant guidelines of proclamation of the Standing Committee of Hubei People’s Congress (PSCH no. 5), People’s Republic of China. A total of 216 28-day-old Duroc×Landrace×Yorkshire weaned piglets were randomly divided into three groups (control, antibiotic, and probiotic groups) with six repetitions (12 piglets each) per group having equal numbers of males and females. The control group was fed with complete ration (corn-soybean meal-dairy ration) (Table 1) according to the U.S. NRC (56) feeding standard with free availability of drinking water. For the antibiotic group, 300 ppm oxytetracycline calcium was added to the complete ration, while the probiotic group was supplemented with 5 × 10^9^ CFU/kg Bacillus amyloliquefaciens on the basis of complete ration. The growth of each piglet was recorded every day throughout the 14-day trial.

**TABLE 1:** The composition and ingredients in the experimental diets^*a*^

Diet component	Percentage
Ingredient	
Corn	22.460
Expanded corn	20.000
Expanded soybean 34.5	6.000
45 soybean meal (protein 45.5)	19.000
Whey powder	15.000
Milk powder	7.500
Sucrose	5.000
Soybean oil	1.500
Calcium formate	0.700
Mono-dicalcium phosphate	0.540
Sodium chloride	0.350
98 lysine	0.580
dl-methionine	0.350
l-threonine	0.260
l-tryptophan	0.100
l-valine	0.160
Premix	0.500
Total %	100.000
Nutrient	
Moisture	9.63
Crude protein	18.50
Crude fat	6.05
Crude ash	6.42
Crude fiber	2.59
Calcium	0.60
Total phosphorus	0.51
DE(NRC)	3594
SID-Lys	1.35
SID-Met	0.58
SID-Thr	0.84
SID-Trp	0.27
SID-Val	0.87
SID-Ile	0.71

a Premix is provided per kilogram of diet: *V*_A_ 25 00 IU, *V*_D3_ 240 IU, *V*_E_ 20 IU, *V*_K3_ 1.0 mg, *V*_B1_ 2.2 mg, *V*_B2_ 5.0 mg, *V*_B12_ 0.05 mg, biotin 0.3 mg, niacin 35 mg, pantothenic acid 20 mg, Fe 100 mg, Cu 80 mg, Mn 8 mg, Zn 80 mg, Se 0.30 mg, I 0.2 mg.

### Collection and handling of samples.

On the 7th day, 36 piglets from each group were randomly slaughtered. A total of 14.5 mL of venous blood was collected, centrifuged at 3,000 rpm for 10 min to separate serum, and then stored at −20°C. Half of the piglets’ spleen, lymph nodes, duodenum, jejunum, ileum, and cecum were placed in 4% paraformaldehyde, while the other half and the contents of the cecum were stored in sterile EP tubes at −80°C.

### Preparation of seeded Bacillus amyloliquefaciens fluid.

The original strain was taken out from the storage (−80°C) and thawed in a water bath. Afterwards, 100 μL of strain was acquired and then inoculated into a 10 mL LB broth medium. An activated seeded bacterial liquid was obtained after incubation (until a large amount of precipitation) on a shaker at 37°C with continuous subcultures up to three generations.

### Probiotic enrichment culture medium.

We specially formulated the culture medium of Bacillus amyloliquefaciens for its characteristics. The main components of the medium were cornmeal (12 g/liter), sucrose (9 g/L), soybean meal (20 g/L), fish meal (15 g/L), potassium dihydrogen phosphate (2 g/L), magnesium sulfate (10 g/L), and calcium carbonate (2 g/L).

### Probiotic growth assay.

The 1% (vol/vol) Bacillus amyloliquefaciens fluid was seeded into LB medium and then kept at 37°C. Then, OD value at 600 nm was measured each hour with a blank LB medium. The growth curve of bacteria was drawn with OD value as ordinate and time in abscissa.

### Probiotic survival assay: acid resistance assay.

LB culture medium with different pH values (1.0, 2.0, 3.0, 4.0) was prepared. The seeded Bacillus amyloliquefaciens fluid was inoculated with 1% (vol/vol) physiological saline at different pH values. The number of viable probiotics was counted and diluted in equal proportions after 3 h of incubation at 37°C. Then, the viable probiotics were inoculated in LB medium. A normal (pH = 7.0) LB culture medium was used as a control group in this assay.

The survival rate was calculated as follows.

$$\mathrm{survival rate}\left(\text{\%}\right)=\frac{N_{1}}{N_{0}}\times 100\text{\%}$$

where *N*_1_ is the number of probiotics in the treatment group and *N*_0_ is the number of probiotics in the control group.

**Bile salt resistance assay.** LB culture medium with different bile salt concentrations (0.1%, 0.2%, 0.3%, and 0.6%) was prepared. The seeded Bacillus amyloliquefaciens fluid was inoculated with physiological saline 2% (vol/vol) at different bile salt concentrations. After incubation at 37°C for 3 h, the fluid was diluted in equal proportion and inoculated in an LB agar medium. The number of viable probiotics was then counted with a normal LB culture medium as a control. The survival rate was calculated as described above.

**High-temperature resistance assay.** The temperature of water was adjusted in advance at 37°C, 45°C, 55°C, 65°C, 75°C, and 85°C. The seeded Bacillus amyloliquefaciens fluid was then inoculated with 1% (vol/vol) physiological saline in a water bath for 30 min at different temperatures. In this assay, normal temperature (37°C) was used as a control. The survival rate was calculated as described above.

### Probiotic inhibition of pathogenic bacteria assay.

Using the Oxford cup method, 100 μL of standard strains of Escherichia coli, Staphylococcus aureus, and *Salmonella* was added to the small holes of LB agar medium. The same procedure was done for Haemophilus parasuis, *Streptococcus*, and *Pasteurella*. The sizes of inhibition zones were then measured after incubation at 37°C for 24 h in the inverted form.

### Antibiotic susceptibility assay.

According to CLSI (57) drug susceptibility standards, the Kirby-Bauer (KB) method was used for drug susceptibility testing in which penicillin, gentamicin, streptomycin, tetracycline, florfenicol, amikacin, doxycycline, enrofloxacin, kanamycin, polymyxin B, clindamycin, ciprofloxacin, furazolidone, neomycin, and cephalexin discs were used. Then, the same procedure as described in the above section was adopted for the measurement of inhibiting zones.

### Biochemical indicators and antioxidant parameters detection.

Blood samples were centrifuged at 3,000 rpm for 20 min to separate the serum. The values of total protein (TP), albumin (ALB), calcium (Ca), and phosphorus (P) were then determined. Antioxidant index of total superoxide dismutase (T-SOD) and glutathione peroxidase (GSH-Px) was detected according to kit instructions (Nanjing Jiancheng Bioengineering Institute). A semiautomatic biochemical detector recorded the absorbance of the liquid for each index.

### H&E staining.

After the slaughter, the fresh spleen, mesenteric lymph nodes, duodenum, jejunum, and ileum of weaned piglets were washed with normal saline to remove the blood and other contents. Then, tissues were cut (3 to 5 cm) and fixed with 4% paraformaldehyde for 24 h. A dehydration was performed with 70%, 80%, 90%, and 95% ethanol for 2, 2, 1, and 1 h. Anhydrous ethanol for 1 h was used further. Then, after being transparent with xylene, the tissue was immersed in liquid wax for embedding. The slice thickness of the microtome was about 5 μm. The slices were attached to the glass slide and allowed to dry at 37°C for 12 h, H&E stained, dried, fixed with neutral resin, and then covered up with a cover glass at the end.

### DNA extraction and RT-qPCR.

According to the manufacturer’s instructions, microbial community genomic DNA was extracted from the contents of the cecum and ileum using the BIOG kit (BAIDAI, China). Simply, 200 μL of the intestinal contents was added with 1 mL of phosphate-buffered saline (PBS) and centrifuged at 500 rpm for 5 min to get the supernatant. Then, centrifugation was performed at 12,000 rpm for 5 min to get the pellet. Then, 200 μL of lysis solution I and 200 μL of lysis solution II with 20 μL of the digestion solution were added. Finally, 500 μL of eluent was added to collect the total DNA into the centrifuge tube. Before RT-qPCR, DNA concentration and purity were determined by NanoDrop2000. The specific primers for TL106 (forward 5′-AGCAACGCGAAGAACCTTAC-3′; reverse 5′-ATTTGACGTCATCCCCACCT-3′) were derived from the NCBI database (55), and RT-qPCR was performed by 50 μL system (double-distilled water [ddH_2_O] 16 μL, SYBR PCR master mix 25 μL, forward primer [10 μM] 2 μL, reverse primer [10 μM] 2 μL, DNA 5 μL) through TOYOBO kit. Dissociation curves were obtained to ensure the specificity of each RT-qPCR.

### 16S RNA sequencing for the microbial diversity of cecum.

According to the manufacturer’s instructions, microbial community genomic DNA was extracted from cecal contents using the FastDNA SPIN kit soil (Mpbio Bio-Tek, USA). The DNA extraction quality was detected using 1% agarose gel, while DNA concentration and purity were determined by NanoDrop2000. 338F (5′-ACTCCTACGGGAGGCAGCAG-3′) and 806R (5′-GGACTACHVGGGTWTCTAAT-3′) for 16S rRNA gene V3-V4 variable region were amplified by PCR. The amplification procedure was as follows: 95°C predenaturation for 3 min, 27 cycles (95°C denaturation for the 30 s, 55°C annealing for 30 s, 72°C extension for 30 s), then 72°C stable extension for 10 min. Finally, samples were stored at 4°C (PCR instrument: ABIGeneAmp 9700). The PCR system was as follows: 5× TransStartFastPfu buffer 4 μL, 2.5 mM deoxynucleoside triphosphates (dNTPs) 2 μL, forward primer (5 μM) 0.8 μL, reverse primer (5 μM) 0.8 μL, TransStartFastPfu DNA polymerase 0.4 μL, template DNA 10 ng, up to 20 μL. Then, 0.8 times the volume of magnetic beads (Vazyme VAHTSTM DNA Clean Beads) were added to 20 μL PCR product, which further absorbed on a magnetic stand for 5 min after shaking. Supernatant was collected that was added in 20 μL 0.8-fold magnetic bead washing solution. The samples were kept at room temperature for 5 min after 80% ethanol (200 μL) was added. For the complete evaporation of alcohol, the 25 μL elution buffer was used. For this purpose, PCR tubes were placed on the adsorption rack for 5 min. The sequencing library was prepared using TruSeq Nano DNA LT library prep kit from Illumina. Before sequencing, a quality inspection was performed on the Agilent Bioanalyzer, using the Agilent high sensitivity DNA kit. The qualified library had only a single peak with no linker. After that, Quant-iT PicoGreen dsDNA assay kit was used to quantify the library on the Promega QuantiFluor fluorescence quantification system.

### Statistical analysis.

All experiments were performed at least five times. GraphPad Prism 7 software was used for statistical analyses. The differences between the groups were analyzed using Student’s *t* test, one-way analysis of variance, and Dunnett’s multiple-comparison test, while a *P* value of <0.05 was considered statistically significant.
